# Comparing cost and print time estimates for six commercially-available 3D printers obtained through slicing software for clinically relevant anatomical models

**DOI:** 10.1186/s41205-020-00091-4

**Published:** 2021-01-06

**Authors:** Joshua V. Chen, Alan B. C. Dang, Alexis Dang

**Affiliations:** 1 Department of Orthopaedic Surgery, University of California, San Francisco, USA; 2 CA Department of Surgery, San Francisco VA Health Center, Orthopaedic Section, San Francisco, USA

**Keywords:** 3D printing, FDM, SLA, PolyJet, Optimization, Cost, Print time, Clinical utility

## Abstract

**Background:**

3D printed patient-specific anatomical models have been applied clinically to orthopaedic care for surgical planning and patient education. The estimated cost and print time per model for 3D printers have not yet been compared with clinically representative models across multiple printing technologies. This study investigates six commercially-available 3D printers: Prusa i3 MK3S, Formlabs Form 2, Formlabs Form 3, LulzBot TAZ 6, Stratasys F370, and Stratasys J750 Digital Anatomy.

**Methods:**

Seven representative orthopaedic standard tessellation models derived from CT scans were imported into the respective slicing software for each 3D printer. For each printer and corresponding print setting, the slicing software provides a print time and material use estimate. Material quantity was used to calculate estimated model cost. Print settings investigated were infill percentage, layer height, and model orientation on the print bed. The slicing software investigated are Cura LulzBot Edition 3.6.20, GrabCAD Print 1.43, PreForm 3.4.6, and PrusaSlicer 2.2.0.

**Results:**

The effect of changing infill between 15% and 20% on estimated print time and material use was negligible. Orientation of the model has considerable impact on time and cost with worst-case differences being as much as 39.30% added print time and 34.56% added costs. Averaged across all investigated settings, horizontal model orientation on the print bed minimizes estimated print time for all 3D printers, while vertical model orientation minimizes cost with the exception of Stratasys J750 Digital Anatomy, in which horizontal orientation also minimized cost. Decreasing layer height for all investigated printers increased estimated print time and decreased estimated cost with the exception of Stratasys F370, in which cost increased. The difference in material cost was two orders of magnitude between the least and most-expensive printers. The difference in build rate (cm^3^/min) was one order of magnitude between the fastest and slowest printers.

**Conclusions:**

All investigated 3D printers in this study have the potential for clinical utility. Print time and print cost are dependent on orientation of anatomy and the printers and settings selected. Cost-effective clinical 3D printing of anatomic models should consider an appropriate printer for the complexity of the anatomy and the experience of the printer technicians.

**Supplementary Information:**

The online version contains supplementary material available at 10.1186/s41205-020-00091-4.

## Background

3D printing technology is becoming increasingly involved in the current era of delivering medical care and is being applied towards creating personalized prosthetics, 3D printed surgical instruments, medical student and resident education, and patient-specific anatomical models to help guide surgeons preoperatively and intraoperatively [1–9]. Previous studies have demonstrated the possibility of producing cost-effective yet robust 3D printed surgical retractors that far exceed the threshold for clinically excessive retraction in the operating room even after autoclave sterilization [6, 10]. Additionally, literature supports significant cost savings due to reduced operating room time associated with the use of 3D printed patient anatomical models in surgical applications [11]. 3D printing has also become especially relevant due to the COVID-19 pandemic in 2020, where 3D printing was employed to combat shortages in essential medical equipment including ventilator components, N95 respirators, nasopharyngeal collection swabs, and splash-proof face shields [12–19]. Therefore, as 3D printing technologies integrate into medical care, it becomes important to understand and optimize the time and cost needed to produce clinically relevant 3D prints. This knowledge may potentially be applied to time-sensitive fracture care [20, 21].

The three common 3D printing techniques investigated in this study are material extrusion, vat polymerization, and material jetting. These are alternatively known as fused deposition modeling (FDM), stereolithography (SLA), and PolyJet, respectively. FDM printing is based on the continuous extrusion of a heated thermoplastic from a nozzle, SLA printing is based on the polymerization of resin from a resin vat using ultraviolet (UV) light, and PolyJet is based on the UV light mediated polymerization of liquid photopolymer material administered from an ink-jet, all three of which occur in a layer by layer process [1, 9, 22].

The aim of this paper is to evaluate the time and cost required to print seven orthopaedic disease models obtained from anonymized CT scans varied by 3D printer, model orientation on the print bed, and layer height and infill percentage, if applicable. Specifically, this study will investigate six commercially-available 3D printers: Prusa i3 MK3S, Formlabs Form 2, Formlabs Form 3, LulzBot TAZ 6, Stratasys F370, and Stratasys J750 Digital Anatomy.

## Methods

### Standard tessellation language (STL) file preparation

**Fig. 1:**
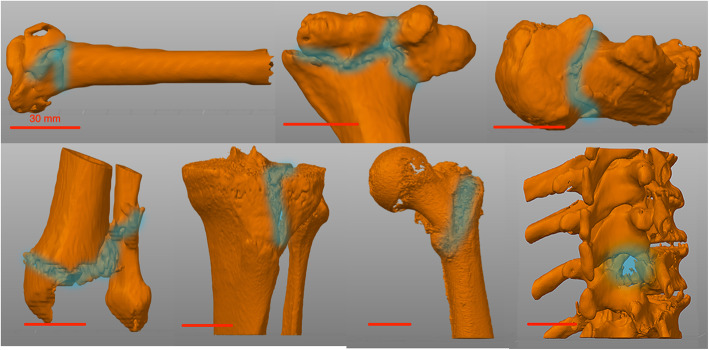
Top row, left to right: distal radial fracture, distal humeral fracture, calcaneal fracture. Bottom row, left to right: pilon fracture, tibial plateau fracture, femoral IT fracture, spine tumor. Lesions are highlighted on the image

Seven STLs of orthopaedic models were derived from anonymized DICOM CT scans of the following disease states: distal radial fracture, distal humeral fracture, calcaneal fracture, spine tumor, pilon fracture, tibial plateau fracture, and femoral intertrochanteric (IT) fracture (Fig. 1).

2D CT scan images were imported into the DICOM viewing software OsiriX MD (Pixmeo SARL, Geneva, Switzerland), segmented to select pertinent anatomy, and exported as a 3D model [23]. For further processing, this 3D model was then imported into the software Autodesk Meshmixer V.3.5, where a triangle mesh of the model was created, thereby creating a more printer-friendly model with reduced surface roughness and model noise [23]. These models are not solid, and are created with accurate cortical thickness. This model was exported from Autodesk Meshmixer as an STL file.

### Printers and slicing software

Orthopaedic models in the form of STL files were then imported into the slicing software for each 3D printer, which is capable of providing print time and material use estimates of each model after adding supports, given orientation and infill percentage; all other default print settings, including print speed, first layer speed, infill speed, wall speed, number of perimeters, and travel speed, were maintained (Table 1, 2) (Appendix B). In this study, infill can be changed only for FDM printers.

**Table 1:** 3D printer details and costs

3D printer	Printing technique	Materials	Materials cost	Build volume (L x W x H)	Slicing software	Cost of preassembled printer (USD)	Additional post-processing materials
Formlabs Form 2	SLA	Clear resin V4 + consumable resin tank	$149/L + $60/2L	145 x 145 x 175 mm	PreForm 3.4.6	$3,499	Isopropyl Alcohol: $17.75/gal Form Wash: $499 Form Cure: $699
Formlabs Form 3	SLA	Clear resin V4 + consumable resin tank	$149/L + $60/2L	145 x 145 x 185 mm	PreForm 3.4.6	$3,499	Isopropyl Alcohol: $17.75/gal Form Wash: $499 Form Cure: $699
LulzBot TAZ 6	FDM	PLA (generic)	$20/kg	280 x 280 x 250 mm	Cura LulzBot Edition 3.6.20	$2,500	None
Prusa i3 MK3S	FDM	PLA (generic)	$20/kg	250 x 210 x 200 mm	PrusaSlicer 2.2.0	$999	None
Stratasys F370	FDM	F123 ABS + F123 QSR support	$187/60 in^3^ + $182/60 in^3^	355 x 254 x 355 mm	GrabCAD Print 1.43	$60,000	None
Stratasys J750 Digital Anatomy	PolyJet	Liquid photopolymer + SUP706 support	$302.50 - $432.26/kg + $130/kg	490 x 390 x 200 mm	GrabCAD Print 1.43	$300,000	None

**Table 2:** Slicing software print settings

Slicing software (3D Printer)	Miscellaneous print settings
Cura LulzBot Edition 3.6.20 (LulzBot TAZ 6)	Experimental: tree support; all other default print settings
GrabCAD Print - Version 1.43 (Stratasys F370)	Infill: sparse; all other default print and support settings
GrabCAD Print - Version 1.43 (Stratasys J750 Digital Anatomy)	Default print and support settings
PreForm 3.4.6 (Formlabs Form 2)	Material changed to Resin Clear V4; all other default print and support settings
PreForm 3.4.6 (Formlabs Form 3)	Material changed to Resin Clear V4; all other default print and support settings
PrusaSlicer 2.2.0 (Prusa i3 MK3S)	Default print and support settings

The estimated cost was subsequently calculated, taking into account slicing software estimated material use and consumables cost, the latter of which only includes consumable resin tanks for Formlabs Form 2 and Form 3 printers. For Form 2 and Form 3, model cost was calculated by multiplying the slicer estimated material use by the cost per milliliter (mL) of resin added to the cost of consumable resin tank per mL. For all other 3D printers, model cost was calculated by multiplying the slicer estimated material use by the cost per unit of material. Additional post-processing accessories and materials have not been added to the estimated cost, and are accounted for separately as they are not contingent on material use.

Models were automatically centered on the build plate in each slicing software, with the exceptions of PreForm 3.4.6 for FormLabs Form 3 and GrabCAD Print – Version 1.43 for Stratasys J750 Digital Anatomy in which models were automatically placed on the corner of the build plate. The model location on the build plate resulted in no or insignificant changes to print time and material use, with the exception of Stratasys J750 Digital Anatomy, in which model location has considerable impact on estimated print time and material use. The Stratasys J750 Digital Anatomy template in GrabCAD Print – Version 1.43 automatically places models on the print bed such that print time and material use is minimized.

### Investigated print settings

For FDM 3D printers, we investigated the common infill percentages of 15% and 20% to assess for differences in print time and material use (Table 3). When a layer height of 0.01 in, or 0.254 mm, is selected, Stratasys F370 requires a minimum infill of 17%, and therefore we only investigated models with 20% infill for this setting. Furthermore, a layer height of 0.007 in, or 0.1778 mm, requires a minimum infill of 23%, and therefore estimates could not be obtained for this layer height.

**Table 3:** Incomplete data due to 3D printer or slicing software limitations

3D Printer	Infill percentage	Layer height	Incomplete data	Notes
Formlabs Form 2	N/A	0.05 mm, 0.10 mm	Femoral IT fracture model in horizontal and 45 degrees orientation does not fit on build plate.	0.10 mm is maximum layer height.
Formlabs Form 3	N/A	0.05 mm, 0.10 mm	Femoral IT fracture model in horizontal and 45 degrees orientation does not fit on build plate.	0.10 mm is maximum layer height.
LulzBot TAZ 6	15%, 20%	0.20 mm, 0.30 mm, 0.38 mm	N/A	0.38 mm layer height is the default setting. Slicing software is unable to slice at layer heights less than 0.19 mm.
Prusa i3 MK3S	15%, 20%	0.15 mm, 0.20 mm, 0.30 mm	At 0.30 mm layer height, spine tumor model indicates an error that empty layers were detected.	N/A
Stratasys F370	15%, 20%	0.01 in (0.254 mm), 0.013 in (0.3302 mm)	15% infill at 0.01 in (0.254 mm) layer height could not be sliced.	0.01 in (0.254 mm) layer height requires minimum 17% infill. 0.007 in (0.1778 mm), not investigated, requires minimum 23% infill.
Stratasys J750 Digital Anatomy	N/A	N/A	N/A	Option of choosing High Quality, High Mix, or High Speed.

Slicing software for SLA technology does not have the option of adjusting infill, as the printing and curing process would result in unpolymerized resin becoming trapped within the model. The slicing software for Stratasys J750 Digital Anatomy does not have the option for changing infill; the layer height for the High Quality setting is preset to 0.014 mm, and the layer height for both the High Mix and High Speed setting is preset to 0.027 mm [24].

For all printers, this study will investigate how changes in layer height affect estimated print time and cost.

### Model orientation on the print bed

**Fig. 2:**
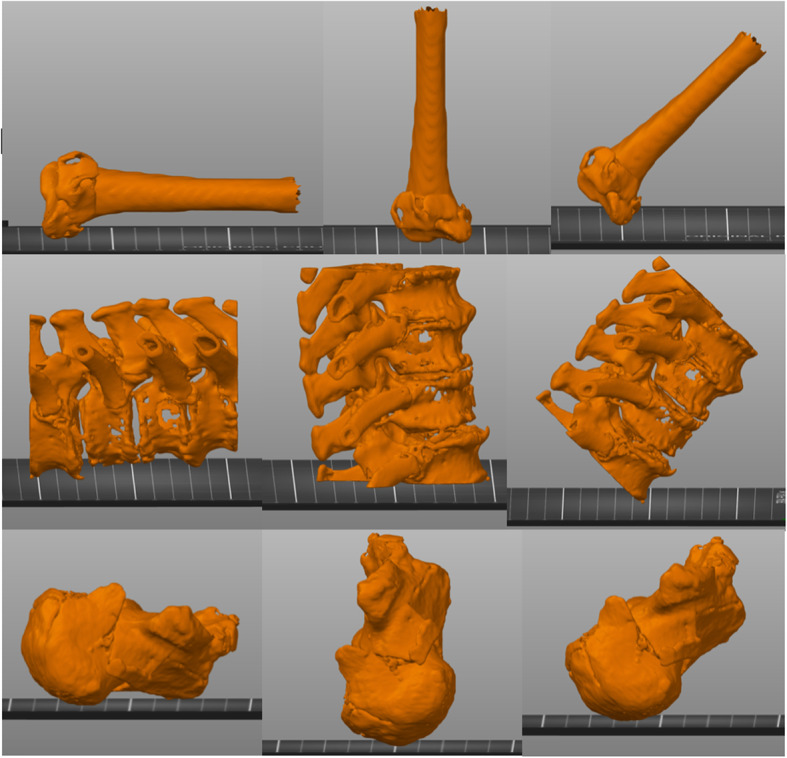
Top row, left to right: Distal radial fracture model horizontal, vertical, 45 degrees. Middle row, left to right: Spine tumor model horizontal, vertical, 45 degrees. Bottom row, left to right: Calcaneal fracture horizontal, vertical, 45 degrees

3D models generated from a CT scan are oriented relative to the patient’s position in the scanner. To assess the effect of model orientation on the build plate on estimated print time and material use, three orientations were defined (Fig. 2).

Horizontal: For long bone models, the long axis is oriented parallel to the build plate. The calcaneal fracture model is oriented in anatomical position. The long axis of the spine tumor model is oriented parallel to the build plate with the spinous processes superior and vertebral bodies inferior.

Vertical: For long bone models, the long axis of the print is oriented perpendicular to the build plate, with the diaphysis oriented superior to the epiphysis. For the calcaneal fracture model, the posterior calcaneus would be oriented inferior to the anterior calcaneus in the axis perpendicular to the build plate. The spine tumor model was maintained in the anatomical position of the patient.

45 degrees: For all models, the process of orientation identical to the vertical orientation, with an additional 45-degree deviation towards the build plate.

### Data collection and interpretation

For each of seven orthopaedic models across three orientations on the print bed, estimated print time and material use were recorded from the slicing software for each printer and corresponding print setting. For some printers and settings, not all models could be sliced, and therefore print time and material use estimates could not be obtained (Table 3). We have accounted for this when interpreting data by omitting incomplete data equally across all compared datasets. Interventions were structured as single setting changes, and the effects on estimated cost and print time were evaluated by the percentage change of these values following intervention.

## Results

### Effect of infill percentage on estimated print time and model cost

The following percentage comparisons for estimated print time were calculated by averaging the estimated print time for each individual model over three orientations, then summing the averaged estimated print time for all seven models for a specific printer and setting. For each layer height, the ratio of the sum after intervention and the sum prior to intervention is taken. These ratios were subsequently averaged to yield the final value. The same process is used to calculate percentage comparisons of estimated cost.

In a comparison between the Prusa i3 MK3S, Stratasys F370, and LulzBot TAZ 6, we have found the percentage increase in print time when increasing infill from 15% to 20% to be 1.01%, 0.60%, and 1.36%, respectively; we have also found the average percentage increase in model cost when increasing infill from 15% to 20% to be 1.71%, 0.19%, and 1.32%, respectively (Table 4).

**Table 4:** Quantifying average percentage change in estimated print time and cost following changes in infill or layer height

3D printer and setting	Intervention	Average percentage change in estimated print time	Average percentage change in estimated cost
Prusa i3 Mk3S	Increasing infill from 15% to 20%	+ 1.01%	+ 1.71%
Stratasys F370	Increasing infill from 15% to 20%	+ 0.60%	+ 0.19%
LulzBot TAZ 6	Increasing infill from 15% to 20%	+ 1.36%	+ 1.32%
Prusa i3 Mk3S, 20% infill	Decreasing layer height from 0.30 mm to 0.20 mm	+ 11.72%	- 2.92%
Prusa i3 Mk3S, 20% infill	Decreasing layer height from 0.30 mm to 0.15 mm	+ 48.04%	- 8.25%
Prusa i3 Mk3S, 15% infill	Decreasing layer height from 0.30 mm to 0.20 mm	+ 11.37%	- 3.13%
Prusa i3 Mk3S, 15% infill	Decreasing layer height from 0.30 mm to 0.15 mm	+ 46.70%	- 8.13%
Stratasys F370, 20% infill	Decreasing layer height from 0.013 in (0.3302 mm) to 0.01 in (0.254 mm)	+ 28.73%	+ 10.29%
LulzBot TAZ 6, 20% infill	Decreasing layer height from 0.38 mm to 0.30 mm	+ 24.27%	- 0.73%
LulzBot TAZ 6, 20% infill	Decreasing layer height from 0.38 mm to 0.20 mm	+ 69.59%	- 9.81%
LulzBot TAZ 6, 15% infill	Decreasing layer height from 0.38 mm to 0.30 mm	+ 24.54%	- 1.03%
LulzBot TAZ 6, 15% infill	Decreasing layer height from 0.38 mm to 0.20 mm	+ 70.58%	- 9.53%
Formlabs Form 2	Decreasing layer height from 0.10 mm to 0.05 mm	+ 59.52%	- 0.75%
Formlabs Form 3	Decreasing layer height from 0.10 mm to 0.05 mm	+ 65.79%	- 0.62%
Stratasys J750 Digital Anatomy, High Mix (0.027 mm layer height)	Changing print setting to High Quality (0.014 mm layer height)	+ 77.10%	+ 41.77%
Stratasys J750 Digital Anatomy, High Speed (0.027 mm layer height)	Changing print setting to High Quality (0.014 mm layer height)	+ 228.98%	+ 39.53%
Stratasys J750 Digital Anatomy, High Speed (0.027 mm layer height)	Changing print setting to High Mix (0.027 mm layer height)	+ 85.76%	- 1.58%

Tabulated print settings investigated in this study by printer

### Effect of layer height on estimated print time and model cost for FDM 3D printers

The following percentage comparisons for estimated print time were calculated by averaging the estimated print time for each individual model over three orientations, then summing the average estimated print time for all seven models for a specific printer and setting. The ratio of the sum after intervention and the sum prior to intervention is taken to yield the final value. The same process is used to calculate percentage comparisons of estimated cost.

For Prusa i3 MK3S at 20% infill, decreasing layer height from 0.30 mm to 0.20 mm increased estimated print time by 11.72% and decreased estimated cost by 2.92%, and decreasing layer height from 0.30 mm to 0.15 mm increased estimated print time by 48.04% and decreased estimated cost by 8.25% (Table 4). For Prusa i3 MK3S at 15% infill, decreasing layer height from 0.30 mm to 0.20 mm increased estimated print time by 11.37% and decreased estimated cost by 3.13%, and decreasing layer height from 0.30 mm to 0.15 mm increased estimated print time by 46.70% and decreased estimated cost by 8.13% (Table 4).

For Stratasys F370 at 20% infill, decreasing layer height from 0.013 in, or 0.3302 mm, to 0.01 in, or 0.254 mm, increased estimated print time by 28.73% and increased estimated cost by 10.29% (Table 4).

For Stratasys J750 Digital Anatomy, layer heights are preset depending on the selected print setting. Changing the High Mix print setting (0.027 mm layer height) to the High Quality print setting (0.014 mm layer height) increased the estimated print time by 77.10% and increased estimated cost by 41.77% (Table 4). Changing the High Speed print setting (0.027 mm layer height) to the High Quality print setting increased the estimated print time by 228.98% and increased estimated cost by 39.53% (Table 4). Changing the High Speed print setting to the High Mix print setting increased estimated print time by 85.76% and decreased estimated cost by 1.58%, despite unchanged layer height (Table 4).

For LulzBot TAZ 6 at 20% infill, decreasing layer height from 0.38 mm, the default setting, to 0.30 mm increased estimated print time by 24.27% and decreased estimated cost by 0.73%, and decreasing layer height from 0.38 mm to 0.20 mm increased estimated print time by 69.59% and decreased estimated cost by 9.81% (Table 4). For LulzBot TAZ 6 at 15% infill, decreasing layer height from 0.38 mm to 0.30 mm increased estimated print time by 24.54% and decreased estimated cost by 1.03%, and decreasing layer height from 0.38 mm to 0.20 mm increased estimated print time by 70.58% and decreased estimated cost by 9.53% (Table 4).

### Effect of layer height on estimated print time and model cost for SLA 3D printers

A process identical to that used to compare layer heights for FDM printers was used to calculate the following percentage comparisons.

For Formlabs Form 2, decreasing layer height from 0.10 mm to 0.05 mm increased estimated print time by 59.52% and decreased estimated cost by 0.75% (Table 4).

For Formlabs Form 3, decreasing layer height from 0.10 mm to 0.05 mm increased estimated print time by 65.79% and decreased estimated cost by 0.62% (Table 4).

### Effect of model orientation on the print bed on estimated print time and model cost

Comparisons with Prusa i3 MK3S 0.30 mm have taken into account incomplete spine tumor model data by equal omission across all three orientations. Spine tumor model data is present for all other datasets. For comparisons between Formlabs Form 2 and Form 3, the femoral IT fracture model has been omitted due to the slicer being unable to provide estimates.

The following percentage comparisons for estimated print time were calculated by summing the total estimated print time for all models for all printers and settings by orientation, yielding an aggregate estimated print time for each orientation. Ratios comparing different aggregate print times by orientation were calculated, yielding a percentage change. The same process is used to calculate percentage comparisons of estimated cost.

For the FDM 3D printers Prusa i3 MK3S, Stratasys F370, and LulzBot TAZ 6, using only data with 20% infill due to the negligible difference in the estimated cost and print time between 15% and 20% infill, the orientation that minimized estimated print time on average was horizontal, with vertical and 45 degrees taking 1.06% and 13.88% longer to print than horizontal, respectively; the orientation that minimized estimated cost on average was vertical, with horizontal and 45 degrees costing 4.84% and 14.14% more than vertical, respectively (Table 5)**.**

**Table 5:** Quantifying average percentage change in estimated print time and cost following changes in model orientation on the print bed

3D printing technology	Intervention	Average percentage change in estimated print time	Average percentage change in estimated cost
FDM	Changing model orientation from horizontal to vertical	+ 1.06%	- 4.62%
FDM	Changing model orientation from horizontal to 45 degrees	+ 13.88%	+ 8.87%
FDM	Changing model orientation from vertical to horizontal	- 1.05%	+ 4.84%
FDM	Changing model orientation from vertical to 45 degrees	+ 12.69%	+ 14.14%
SLA	Changing model orientation from horizontal to vertical	+ 16.63%	- 2.84%
SLA	Changing model orientation from horizontal to 45 degrees	+ 22.92%	+ 4.13%
SLA	Changing model orientation from vertical to horizontal	- 14.26%	+ 2.92%
SLA	Changing model orientation from vertical to 45 degrees	+ 5.39%	+ 7.17%
PolyJet	Changing model orientation from horizontal to vertical	+ 28.94%	+ 15.79%
PolyJet	Changing model orientation from horizontal to 45 degrees	+ 39.30%	+ 34.56%

For the SLA 3D printers Formlabs Form 2 and Form 3, the orientation that minimized estimated print time on average was horizontal, with vertical and 45 degrees taking 16.63% and 22.92% longer than horizontal, respectively; the orientation that minimized estimated cost on average was vertical, with horizontal and 45 degrees costing 2.92% and 7.17% more than vertical, respectively (Table 5).

For the PolyJet 3D printer Stratasys J750 Digital Anatomy, the orientation that minimized estimated print time on average was horizontal, with vertical and 45 degrees taking 28.94% and 39.30% longer than horizontal, respectively; the orientation that minimized estimated cost on average was horizontal, with vertical and 45 degrees costing 15.79% and 34.58% more than horizontal, respectively (Table 5).

### Estimated print time and model cost comparison between FDM 3D printers

Prusa i3 MK3S at 0.30 mm layer height has been omitted due to incomplete spine tumor model data. The remaining printers and settings are compared using data for all seven orthopaedic models, with the infill set to 20%.

**Fig. 3:**
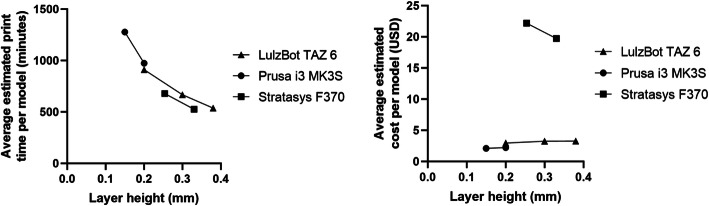
Average estimated print time and cost comparison for FDM 3D printers at 20% infill, excluding Prusa i3 MK3S at 0.30 mm layer height due to incomplete data

To compare FDM printers across different layer heights, the estimated print time and cost of each of the seven orthopaedic models were first averaged across three orientations, then averaged across the seven orthopaedic models, yielding an average estimated print time and cost per model per printer (Fig. 3)**.**

For Prusa i3 MK3S at 0.15 mm layer height, the average estimated print time was 1277.71 min per model and the average estimated cost was $2.12 per model. For Prusa i3 MK3S at 0.20 mm layer height, the average estimated print time was 974.67 min per model and the average estimated cost was $2.24 per model.

For Stratasys F370 at 0.01 in, or 0.254 mm, layer height, the average estimated print time was 679.43 min per model and the average estimated cost was $22.19 per model. For Stratasys F370 at 0.013 in, or 0.3302 mm layer height, the average estimated print time was 526.43 min per model and the average estimated cost was $19.73 per model.

For LulzBot TAZ 6 at 0.20 mm layer height, the average estimated print time was 912.24 min per model and the average estimated cost was $2.96 per model. For LulzBot TAZ 6 at 0.30 mm layer height, the average estimated print time was 668.43 min per model and the average estimated cost was $3.26 per model. For LulzBot TAZ 6 at 0.38 mm layer height, the average estimated print time was 537.90 min per model and the average estimated cost was $3.28 per model.

### Estimated print time and model cost comparison between SLA 3D printers

**Fig. 4:**
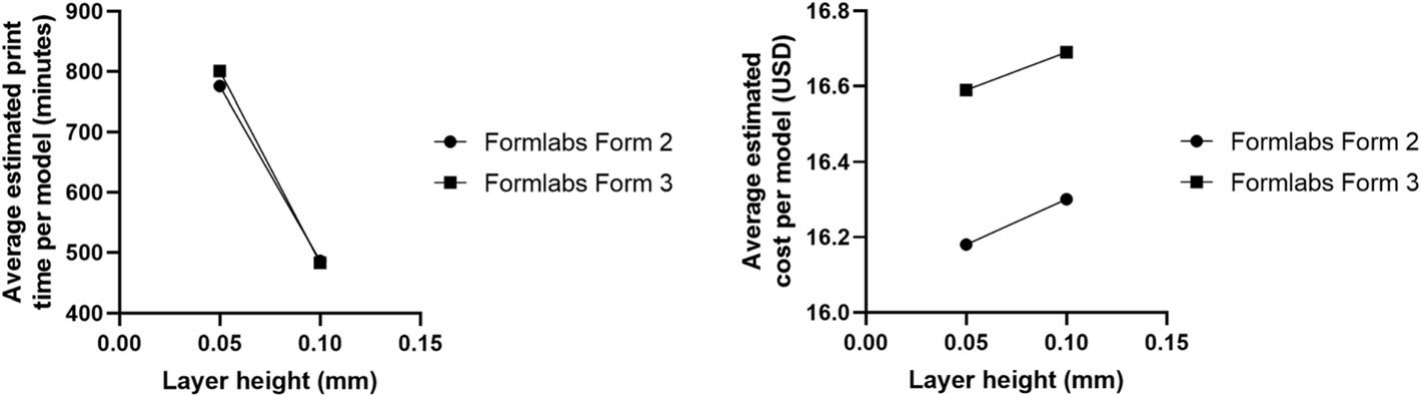
Average estimated print time and cost comparison for SLA 3D printers, excluding the femoral IT fracture model completely due to incomplete data

The following comparisons have omitted the femoral IT fracture model across all SLA printers and settings due to the model being unable to fit on the build plate. A process identical to that used to calculate the average estimated print time and cost per model per printer for FDM printers was used for SLA printers (Fig. 4).

For Formlabs Form 2 at 0.05 mm layer height, the average estimated print time was 776.22 min per model and the average estimated cost was $16.18 per model. For Formlabs Form 2 at 0.10 mm layer height, the average estimated print time was 486.61 min per model and the average estimated cost was $16.30 per model.

For Formlabs Form 3 at 0.05 mm layer height, the average estimated print time was 801.06 min per model and the average estimated cost was $16.59 per model. For Formlabs Form 3 at 0.10 mm layer height, the average estimated print time was 483.17 min per model and the average estimated cost was $16.69 per model.

### Estimated print time and model cost for a PolyJet 3D printer

**Fig. 5:**
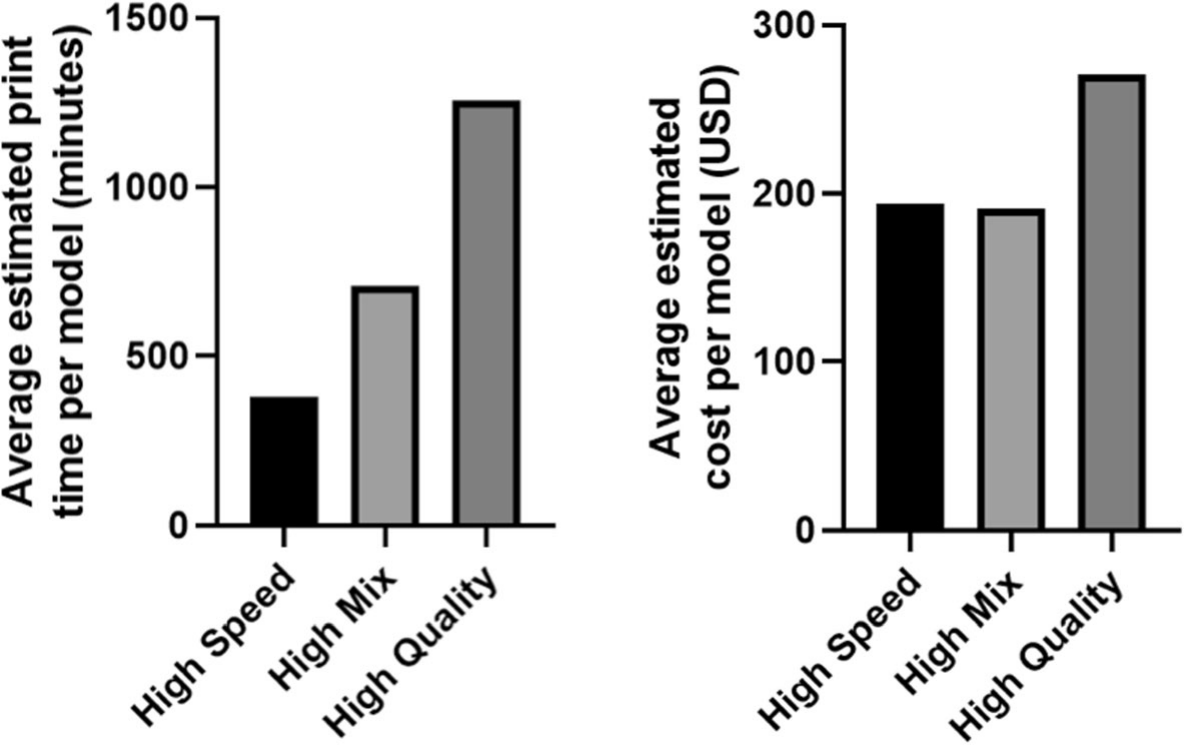
Average estimated print time and cost comparison for different print settings for Stratasys J750 Digital Anatomy

**Fig. 6:**
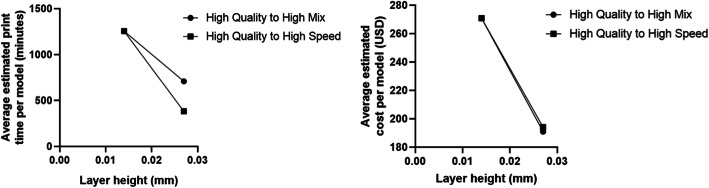
Average estimated print time and cost comparison when changing the print setting from High Quality to High Mix and High Quality to High Speed for Stratasys J750 Digital Anatomy

The slicing software for Stratasys J750 Digital Anatomy allows the user to select three print settings: High Speed, High Mix, and High Quality (Fig. 5). A process identical to that used to calculate the average estimated print time and cost per model per printer for FDM printers was used (Figs. 5, 6).

For the High Speed print setting, the average estimated print time was 381.57 min per model and the average estimated cost was $194.04 per model. For the High Mix print setting, the average estimated print time was 708.81 min per model and the average estimated cost was $190.98 per model. For the High Quality print setting, the average estimated print time was 1255.29 min per model and the average estimated cost was $270.75 per model.

### Estimated print time and model cost comparison for all 3D printers

To ensure a fair comparison, the following print time and cost comparisons have omitted the spine tumor and femoral IT fracture model for all printers and settings due to incomplete data. The FDM printers were set to 20% infill.

The three 3D printers with the lowest achievable estimated print time per model are Stratasys J750 Digital Anatomy, LulzBot TAZ 6, and Stratasys F370 (Table 6).

**Table 6:** Estimated print time per model for all 3D printers and print settings in ascending order, excluding the spine tumor and femoral IT fracture models for all 3D printers and settings

3D Printer	Setting	Estimated print time per model (minutes)
Stratasys J750 Digital Anatomy	High Speed (0.027 mm layer height)	295.73
LulzBot TAZ 6	0.38 mm layer height	341.4
Stratasys F370	0.013 in (0.3302 mm) layer height	382.93
Formlabs Form 3	0.10 mm layer height	403.53
LulzBot TAZ 6	0.30 mm layer height	424.47
Formlabs Form 2	0.10 mm layer height	431.80
Stratasys F370	0.01 in (0.254 mm) layer height	503.07
Stratasys J750 Digital Anatomy	High Mix (0.027 mm layer height)	549.27
LulzBot TAZ 6	0.20 mm layer height	589.93
Prusa i3 MK3S	0.30 mm layer height	619.47
Formlabs Form 3	0.05 mm layer height	674.93
Formlabs Form 2	0.05 mm layer height	688.87
Prusa i3 MK3S	0.20 mm layer height	702.60
Prusa i3 MK3S	0.15 mm layer height	943.07
Stratasys J750 Digital Anatomy	High Quality (0.014 mm layer height)	979.87

To compare the volume of 3D printing material deposited per minute across 3D printers and settings, the estimated volume of the print including support material was divided by the estimated print time, reported as cm^3^/min. This metric, build rate, was averaged across orientations, and then averaged across models. Stratasys J750 Digital Anatomy has the highest average cm^3^/min followed by LulzBot TAZ 6 (Table 7).

**Table 7:** Estimated average build rate (cm^3^ /min) for all 3D printers and print settings in descending order, excluding the spine tumor and femoral IT fracture models for all 3D printers and settings

3D Printer	Setting	Average build rate (cm^**3**^/min)
Stratasys J750 Digital Anatomy	High Mix (0.027 mm layer height)	1.16
Stratasys J750 Digital Anatomy	High Speed (0.027 mm layer height)	1.12
Stratasys J750 Digital Anatomy	High Quality (0.014 mm layer height)	0.90
LulzBot TAZ 6	0.38 mm layer height	0.34
LulzBot TAZ 6	0.30 mm layer height	0.27
Stratasys F370	0.013 in (0.3302 mm) layer height	0.22
Formlabs Form 3	0.10 mm layer height	0.19
Stratasys F370	0.01 in (0.254 mm) layer height	0.19
LulzBot TAZ 6	0.20 mm layer height	0.18
Formlabs Form 2	0.10 mm layer height	0.17
Formlabs Form 3	0.05 mm layer height	0.11
Prusa i3 MK3S	0.30 mm layer height	0.11
Formlabs Form 2	0.05 mm layer height	0.11
Prusa i3 MK3S	0.20 mm layer height	0.09
Prusa i3 MK3S	0.15 mm layer height	0.07

For articular fracture models, the desktop FDM printers Prusa i3 MK3S and LulzBot TAZ 6 were an order of magnitude lower in cost than the desktop SLA printers, Formlabs Form 2 and Form 3, or industrial FDM printer Stratasys F370 (Table 8).

**Table 8:** Estimated cost per model for all 3D printers and print settings in ascending order, excluding the spine tumor and femoral IT fracture models for all 3D printers and settings

3D Printer	Setting	Estimated cost per model (USD)
Prusa i3 MK3S	0.15 mm layer height	$1.45
Prusa i3 MK3S	0.20 mm layer height	$1.53
Prusa i3 MK3S	0.30 mm layer height	$1.57
LulzBot TAZ 6	0.20 mm layer height	$1.71
LulzBot TAZ 6	0.30 mm layer height	$1.84
LulzBot TAZ 6	0.38 mm layer height	$1.87
Formlabs Form 2	0.05 mm layer height	$13.60
Formlabs Form 2	0.10 mm layer height	$13.76
Stratasys F370	0.013 in (0.3302 mm) layer height	$13.92
Formlabs Form 3	0.05 mm layer height	$13.99
Formlabs Form 3	0.10 mm layer height	$14.07
Stratasys F370	0.01 in (0.254 mm) layer height	$16.44
Stratasys J750 Digital Anatomy	High Mix (0.027 mm layer height)	$140.22
Stratasys J750 Digital Anatomy	High Speed (0.027 mm layer height)	$142.66
Stratasys J750 Digital Anatomy	High Quality (0.014 mm layer height)	$203.43

Although the Prusa i3 Mk3S offered the lowest costs for articular fracture models, the default printing presets resulted in inconsistent printing capabilities in the 0.30 mm Draft mode. The femoral IT fracture model was too large for the capabilities of the Form 2 and Form 3.

The following comparisons include all 7 orthopaedic models and excluded printers and settings with incomplete data. The spine tumor and femoral IT fracture models are print time intensive and cost intensive models, as reflected by increases in average print time and cost per model.

The three 3D printers with the lowest achievable estimated print time per model remain Stratasys J750 Digital Anatomy, Stratasys F370, and LulzBot TAZ 6 (Table 9).

**Table 9:** Estimated print time per model for 3D printers and print settings in ascending order, excluding printers and print settings with incomplete orthopaedic model data

3D Printer	Setting	Estimated print time per model (minutes)
Stratasys J750 Digital Anatomy	High Speed (0.027 mm layer height)	381.57
Stratasys F370	0.013 in (0.3302 mm) layer height	526.43
LulzBot TAZ 6	0.38 mm layer height	537.90
LulzBot TAZ 6	0.30 mm layer height	668.43
Stratasys F370	0.01 in (0.254 mm) layer height	679.43
Stratasys J750 Digital Anatomy	High Mix (0.027 mm layer height)	708.81
LulzBot TAZ 6	0.20 mm layer height	912.24
Prusa i3 MK3S	0.20 mm layer height	974.67
Stratasys J750 Digital Anatomy	High Quality (0.014 mm layer height)	1255.29
Prusa i3 MK3S	0.15 mm layer height	1277.71

Average cm^3^/min was again compared across 3D printers, with Stratasys J750 Digital Anatomy again yielding the highest average cm^3^/min followed by LulzBot TAZ 6 (Table 10).

**Table 10:** Estimated average build rate (cm^3^ /min) for all 3D printers and print settings in descending order, excluding printers and print settings with incomplete orthopaedic 3 model data

3D Printer	Setting	Average build rate (cm^**3**^/min)
Stratasys J750 Digital Anatomy	High Mix (0.027 mm layer height)	1.25
Stratasys J750 Digital Anatomy	High Speed (0.027 mm layer height)	1.07
Stratasys J750 Digital Anatomy	High Quality (0.014 mm layer height)	0.95
LulzBot TAZ 6	0.38 mm layer height	0.36
LulzBot TAZ 6	0.30 mm layer height	0.28
Stratasys F370	0.013 in (0.3302 mm) layer height	0.21
LulzBot TAZ 6	0.20 mm layer height	0.19
Stratasys F370	0.01 in (0.254 mm) layer height	0.18
Prusa i3 MK3S	0.20 mm layer height	0.09
Prusa i3 MK3S	0.15 mm layer height	0.07

The estimated cost per model for Prusa i3 MK3S and LulzBot TAZ 6 remain an order of magnitude lower in cost than Formlabs Form 2 and Form 3 and Stratasys F370, and two orders of magnitude lower than Stratasys J750 Digital Anatomy (Table 11).

**Table 11:** Estimated cost per model for 3D printers and print settings in ascending order, excluding printers and print settings with incomplete orthopaedic model data

3D Printer	Setting	Estimated cost per model (USD)
Prusa i3 MK3S	0.15 mm layer height	$2.12
Prusa i3 MK3S	0.20 mm layer height	$2.24
LulzBot TAZ 6	0.20 mm layer height	$2.96
LulzBot TAZ 6	0.30 mm layer height	$3.26
LulzBot TAZ 6	0.38 mm layer height	$3.28
Stratasys F370	0.013 in (0.3302 mm) layer height	$19.73
Stratasys F370	0.01 in (0.254 mm) layer height	$22.19
Stratasys J750 Digital Anatomy	High Mix (0.027 mm layer height)	$190.98
Stratasys J750 Digital Anatomy	High Speed (0.027 mm layer height)	$194.04
Stratasys J750 Digital Anatomy	High Quality (0.014 mm layer height)	$270.75

## Discussion

### Effect of print settings on estimated print time and model cost

The effect of changing infill between 15% and 20% on estimated print time and cost appears to be negligible, with a maximum percentage change of 1.71% for any print time or cost value across all FDM printers in this study.

For all investigated FDM printers, Prusa i3 MK3S, LulzBot TAZ 6, and Stratasys F370, decreasing layer height increased estimated print time. The inverse relationship between layer height and estimated print time is expected, as increasing layer height reduces the total number of layers required to complete the 3D print, and therefore print time is expected to decrease [25]. For Prusa i3 MK3S and LulzBot TAZ 6, decreasing layer height decreased estimated cost. Increasing layer height reduces the resolution of the print, thereby creating a stair-step effect as layers are deposited; this effect may consume additional 3D printing material as material is extruded outside the boundaries of the model [26]. For Stratasys F370, however, decreasing layer height increased estimated cost. Therefore for Stratasys F370, from slicer estimates, it is both time and cost-effective to increase layer height. The observed magnitude of change in average estimated cost when changing layer height is relatively small compared to the observed magnitude of change in average print time when changing layer height (Fig. 3).

For all investigated SLA printers, Formlabs Form 2 and Form 3, decreasing layer height increased estimated print time and negligibly decreased estimated cost by less than 1%. As with FDM printers, we observed an expected inverse relationship between layer height and print time. A closer inspection of data reveals that the estimated model volume (mL), a measurement directly related to model cost, was not consistently higher for all models when layer height was set to 0.10 mm as compared to 0.05 mm for both Formlabs Form 2 and Form 3 that leads us to conclude that cost is largely unchanged. SLA printers require additional post-processing steps, including model washing with isopropyl alcohol (IPA) and model curing with UV light, which requires an additional 10 min and 15 min respectively per model for the material Clear Resin V4 [27, 28]. The Form Wash post-processing accessory has a 2.3 gal capacity for IPA and can wash up to 200 models before requiring IPA replacement [29, 30]. This adds approximately $0.20 of IPA cost to each model printed on either Formlabs Form 2 or Form 3.

### Effect of model orientation on the print bed on estimated print time and model cost

For all investigated printers, the orientation that minimized estimated print time on average was horizontal, followed by vertical, with 45 degrees orientation being the least time-efficient.

For all investigated printers, except Stratasys J750 Digital Anatomy, the orientation that minimized estimated cost on average was vertical, followed by horizontal, with 45 degrees orientation being the least cost-efficient. For Stratasys J750 Digital Anatomy, the orientation that minimized estimated cost, on average, was horizontal, followed by vertical, with 45 degrees orientation being the least cost-efficient.

Evaluating failure rate based on model orientation on the print bed is outside the scope of this study.

### Estimated print time and model cost comparison between FDM 3D printers

At a given layer height, by extrapolation, Stratasys F370 has a lower estimated print time per model than LulzBot TAZ 6, and the LulzBot TAZ 6 has a lower estimated print time per model than Prusa i3 MK3S (Fig. 3).

The average estimated model costs for Prusa i3 MK3S and LulzBot TAZ 6 are low and comparable with one another. The average estimated model cost for Stratasys F370 is one order of magnitude greater compared to Prusa i3 MK3S and LulzBot TAZ 6.

### Estimated print time and model cost comparison between SLA 3D printers

Formlabs Form 2 and Form 3 are comparable printers with no clear differences in estimated print time or model cost.

### Characterizing estimated print time and cost for PolyJet technology

This study investigates a single PolyJet printer but quantifies estimated print time and cost differences between the Stratasys J750 Digital Anatomy print settings High Speed, High Mix, and High Quality. The corresponding layer heights for these settings were 0.014 mm, 0.027 mm, and 0.027 mm, respectively.

The estimated print time per model is the lowest on the High Speed setting, followed by the High Mix setting. The estimated model costs between High Speed and High Mix are comparable, and both lower than the estimated cost of High Quality. Analysis of layer height reveals a similar trend to FDM and SLA technology, as decreased layer height increased estimated print time. Additionally, average estimated cost was nearly identical for both High Speed and High Mix print settings, both of which have the same preset layer heights.

### Characterizing estimated print time and cost for all 3D printers

Prusa i3 MK3S is a low-cost FDM 3D printer that yields the lowest estimated cost per model but has a high estimated print time with the lowest average build rate. PrusaSlicer 2.2.0 may fail to slice models at a layer height of 0.30 mm.

Stratasys J750 Digital Anatomy is a high-cost PolyJet 3D printer that yields high-resolution prints at a layer height of 0.014 mm or 0.027 mm, and has the lowest estimated print time per model on the High Speed print setting but has a high estimated model cost. This printer has the highest average build rate out of all investigated printers.

The estimated print time and cost for Formlabs Form 2 and Form 3 are comparable, and these SLA 3D printers are able to quickly print high-resolution models. These medium-cost printers yield low estimated print time at 0.10 mm layer height and have medium-range estimated model cost, but require additional time, materials, and accessories for post-processing. Additionally, the build space for Form 2 and Form 3 may be too small for some anatomical models, such as the femoral IT fracture model in this study.

Stratasys F370 is a high-cost FDM 3D printer that yields low estimated print time at a 0.013 in, or 0.3302 mm, layer height and has medium-range estimated model cost.

LulzBot TAZ 6 is a medium-cost FDM 3D printer that yields low estimated print time at a 0.38 mm layer height and low estimated model cost.

### Clinical implications

Current 3D printers have very high resolution that exceeds the current imaging protocol slice thickness requirements and needs for anatomical models. In those instances, lower resolution or faster prints are preferred. Desktop printers offered the lowest costs for models, however certain complex anatomical models require additional user expertise for appropriate orientation due to the risk of obscuring clinically relevant details due to support artifacts. The desktop FDM printers rely on a labor intensive mechanical removal of support structures, whereas the industrial FDM and PolyJet printers allows for a chemical dissolving of support structures with less labor, however the costs and time for support removal was outside of the scope of this study.

### Limitations

We acknowledge that there are many additional 3D printers available on the market that have not been investigated in this study. Additionally, this study only analyzed orthopaedic disease models, and findings may not be generalizable to other solid organ anatomical models.

Assessing pre-processing time, namely the time required to slice a model, is outside the scope of this study as this varies based on computer capabilities. Additionally, assessing post-processing time for 3D printed models, such as support removal, is outside the scope of this study. Post-processing time estimates for SLA 3D printers were obtained directly from the manufacturer’s website. Furthermore, this study does not assess the quality of post-processed 3D printed models, which may include support artifacts or may otherwise be clinically ineffective due to obscured details.

We acknowledge that these prints are simulated on slicing software and have not been validated through physical prints; however, using slicing software is a controlled and reproducible method of obtaining print time and material use estimates. Future steps are required to assess the accuracy of slicing software print time and material use estimates.

The infill percentages selected, 15% and 20%, may be too similar to detect substantial differences in estimated print time and cost. We did not take into account the rate of failure, electricity consumption, 3D printer cost, 3D printer depreciation, or post-processing costs in calculating cost estimates per model, but have included values on 3D printer and post-processing costs.

The additional 3D printing technologies, selective laser sintering (SLS), direct metal laser sintering (DMLS), and Multi-Jet modeling, have not been investigated and is outside the scope of this study [1].

This study is meant to be a preliminary assessment of estimated print time and cost of commercial-available 3D printers through slicing software, and requires further investigation.

## Conclusion

Changing infill between 15% and 20% yields negligible differences in estimated print time and cost. Horizontal model orientation minimizes estimated print time, while vertical model orientation generally minimizes estimated cost with the exception of Stratasys J750 Digital Anatomy, in which horizontal model orientation minimized cost. Decreasing layer height for all 3D printers investigated in this study increased estimated print time and decreased estimated cost with the exception of Stratasys F370, in which estimated cost increased. The Stratasys J750 Digital Anatomy print settings of High Speed and High Mix allows for the reduction of estimated print time and cost.

All investigated printers in this study have the potential for clinical utility. Lower cost desktop 3D printers require additional expertise to minimize the risk of support artifacts obscuring clinically relevant details, and users may encounter slicing software limitations at larger layer heights, build space limitations, and added post-processing labor costs.

Cost-effective clinical 3D printing of anatomic models should consider an appropriate printer for the complexity of the anatomy and the experience of the printer technicians.

## Supplementary Information

**Additional file 1.** Appendix A.

**Additional file 2.** Appendix B.

## Data Availability

Data are available through Appendix A.

## Abbreviations

DMLS: Direct metal laser sintering
FDM: Fused deposition modeling
IPA: Isopropyl alcohol
IT: Intertrochanteric
SLA: Stereolithography
SLS: Selective laser sintering
STL: Standard tessellation language
UV: Ultraviolet

## References

[1] Hoang D, Perrault D, Stevanovic M, Ghiassi A (2016). Surgical applications of three-dimensional printing: a review of the current literature & how to get started. Ann Transl Med.

[2] Garcia J, Yang Z, Mongrain R, Leask RL, Lachapelle K (2018). 3D printing materials and their use in medical education: a review of current technology and trends for the future. BMJ Simul Technol Enhanced Learn.

[3] Paul GM, Rezaienia A, Wen P, Condoor S, Parkar N, King W (2018). Medical applications for 3D printing: recent developments. Mo Med.

[4] Brown C (2017). 3D printing set to revolutionize medicine. Can Med Assoc J.

[5] Lioufas PA, Quayle MR, Leong JC, McMenamin PG (2016). 3D printed models of cleft palate pathology for surgical education: plastic and reconstructive surgery. Global Open.

[6] Chen JV, Dang ABC, Lee CS, Dang ABC (2019). 3D printed PLA Army-navy retractors when used as linear retractors yield clinically acceptable tolerances. 3D Print Med.

[7] Trace AP, Ortiz D, Deal A, Retrouvey M, Elzie C, Goodmurphy C (2016). Radiology’s emerging role in 3-D printing applications in health care. J Am Coll Radiol.

[8] Chepelev L, Wake N, Ryan J, Althobaity W, Gupta A, Arribas E (2018). Radiological Society of North America (RSNA) 3D printing special interest group (SIG): guidelines for medical 3D printing and appropriateness for clinical scenarios. 3D Print Mede.

[9] Mitsouras D, Liacouras P, Imanzadeh A, Giannopoulos AA, Cai T, Kumamaru KK (2015). Medical 3D printing for the radiologist. RadioGraphics.

[10] Chen JV, Tanaka KS, Dang ABC, Dang A (2020). Identifying a commercially-available 3D printing process that minimizes model distortion after annealing and autoclaving and the effect of steam sterilization on mechanical strength. 3D Print Med.

[11] Ballard DH, Mills P, Duszak R, Weisman JA, Rybicki FJ, Woodard PK (2019). Medical 3D printing cost-Savings in Orthopedic and Maxillofacial Surgery: cost analysis of operating room time saved with 3D printed anatomic models and surgical guides. Acad Radiol.

[12] Tino R, Moore R, Antoline S, Ravi P, Wake N, Ionita CN (2020). COVID-19 and the role of 3D printing in medicine. 3D Print Med.

[13] Amin D, Nguyen N, Roser SM, Abramowicz S (2020). 3D printing of face shields during COVID-19 pandemic: a technical note. J Oral Maxillofac Surg.

[14] Flanagan ST, Ballard DH (2020). 3D printed face shields: a community response to the COVID-19 global pandemic. Acad Radiol.

[15] Ishack S, Lipner SR (2020). Applications of 3D printing technology to address COVID-19–related supply shortages. Am J Med.

[16] Erickson MM, Richardson ES, Hernandez NM, Bobbert DW, Gall K, Fearis P (2020). Helmet modification to PPE with 3D printing during the COVID-19 pandemic at Duke University medical center: a novel technique. J Arthroplast.

[17] Cavallo L, Marcianò A, Cicciù M, Oteri G (2020). 3D printing beyond dentistry during COVID 19 epidemic: a technical note for producing connectors to breathing devices. Prosthesis.

[18] Callahan CJ, Lee R, Zulauf KE, Tamburello L, Smith KP, Previtera J (2020). Open development and clinical validation of multiple 3D-printed nasopharyngeal collection swabs: rapid resolution of a critical COVID-19 testing bottleneck. J Clin Microbiol.

[19] Salmi M, Akmal JS, Pei E, Wolff J, Jaribion A, Khajavi SH (2020). 3D printing in COVID-19: productivity estimation of the Most promising open source solutions in emergency situations. Appl Sci.

[20] Bizzotto N, Sandri A, Regis D, Romani D, Tami I, Magnan B (2015). Three-dimensional printing of bone fractures: a new tangible realistic way for preoperative planning and education. Surg Innov.

[21] Brown GA, Firoozbakhsh K, Decoster TA, Reyna JR, Moneim M (2003). Rapid prototyping: the future of trauma surgery?. J Bone Joint Surg.

[22] Bagheri A, Jin J (2019). Photopolymerization in 3D printing. ACS Appl Polymer Mater.

[23] Bücking TM, Hill ER, Robertson JL, Maneas E, Plumb AA, Nikitichev DI (2017). From medical imaging data to 3D printed anatomical models. PLoS One.

[24] Stratasys (2017). Stratasys J750 unleash your imagination with never-before-seen multi-material capabilities printer spec sheets.

[25] Cain P (2020). The impact of layer height on a 3D print.

[26] Günther D, Heymel B, Franz Günther J, Ederer I (2014). Continuous 3D-printing for additive manufacturing. Rapid Prototyp J.

[27] Formlabs Form Wash time settings. https://support.formlabs.com/s/article/Form-Wash-Time-Settings?language=en_US.

[28] Formlabs Form Cure time and temperature settings. https://support.formlabs.com/s/article/Form-Cure-Time-and-Temperature-Settings?language=en_US.

[29] Formlabs When to replace IPA. https://support.formlabs.com/s/article/Measuring-IPAs-Resin-Concentration?language=en_US.

[30] Formlabs Form Wash + Form Cure. https://formlabs.com/wash-cure/.

